# Supplementary material for: Causal Relationship between Aging and Anorexia Nervosa: A White-Matter-Microstructure-Mediated Mendelian Randomization Analysis
Source: Biomedicines. 2024 Aug 16;12(8):1874. doi: 10.3390/biomedicines12081874 (PMC11351342; doi:10.3390/biomedicines12081874)
Supplement: Supplementary file 1 [file biomedicines-12-01874-s001.zip › Supplementary Figure/Figure S7/Single SNP plot of MR analysis for ubm-b-1971 to AN.pdf]

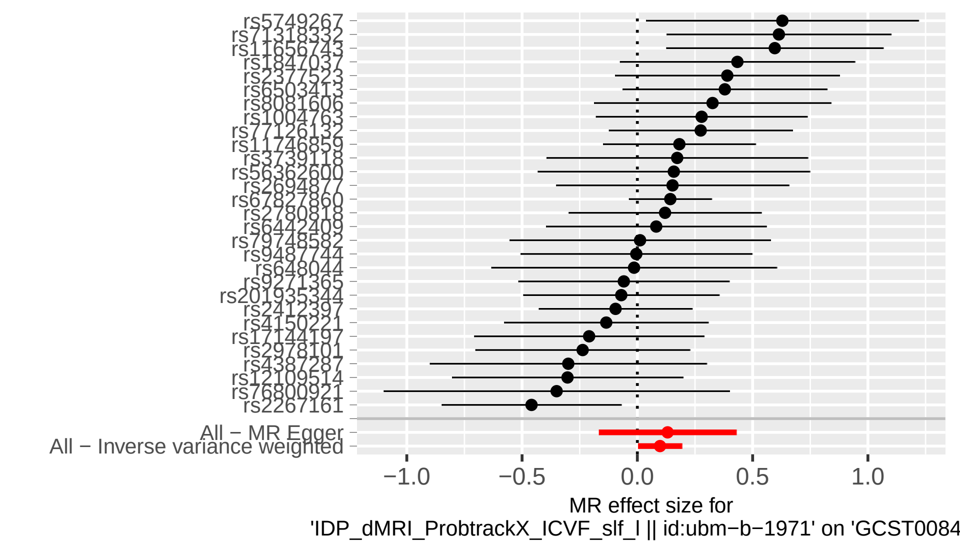
rs5749267
rs71318332
rs11656743
rs1847037
rs2377523
rs6503413
rs8081606
rs1004763
rs77126132
rs11746859
rs3739118
rs56362600
rs2694877
rs67827860
rs2780818
rs6442409
rs79748582
rs9487744
rs648044
rs9271365
rs201935344
rs2412397
rs4150221
rs17144197
rs2978101
rs4387287
rs12109514
rs76800921
rs2267161
All − MR Egger
All − Inverse variance weighted
−1.0
−0.5
0.0
0.5
1.0
MR effect size for
'IDP_dMRI_ProbtrackX_ICVF_slf_l || id:ubm−b−1971' on 'GCST0084
